# EDITORIAL COMMENT: LAPAROSCOPIC SINGLE PORT CYSTOLITHOTOMY USING PNEUMOVESICUM

**DOI:** 10.1590/S1677-5538.IBJU.2016.0337.2

**Published:** 2016

**Authors:** Trushar Patel

**Affiliations:** 1 Department of Urology, University of South Florida, Florida, USA

In an era with robotic partial nephrectomy dominating as the predominate modality to tackle small renal masses, Choiand Bae (1) demonstrate that advanced laparoscopic skill in urology is still alive and well. The difficulty in management with multiple renal tumors is not only nephron preservation, but how to limit the amount of warm ischemia when having to preform multiple resections and renorrhaphies. The utilization of off clamp partial technique in managing smaller and less complex masses allows to maintain manageable ischemia time, obviating the risk of permanent renal damage. While technically challenging, laparoscopic partial nephrectomy can afford all the benefits of minimally invasive surgery similar to robotics but yet without the costs. The challenge will become how do we continue to keep this skill set alive, as more and more training programs preform less laparoscopy and more robotics.

***Trushar Patel, MD*** *Department of Urology* *University of South Florida* *2 Tampa General Circle, STC6* *Tampa, FL 33606, USA* *E-mail: tpatel7@health.usf.edu*
